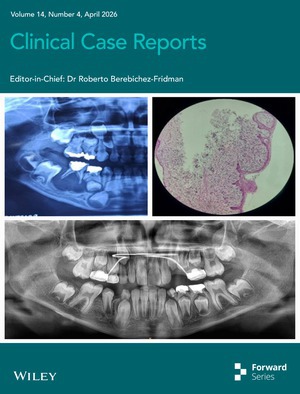# Cover Image

**DOI:** 10.1002/ccr3.72525

**Published:** 2026-04-09

**Authors:** Fahimeh Anbari, Hamid Reza Khalighi, Fatemeh Mashhadiabbas, Zahra Yazdani, Amirmohammad Salamatmanesh

## Abstract

The cover image is based on the article *Radicular Cyst in the Lower Right Primary Tooth and Its Management: A Case Report* by Fahimeh Anbari et al., https://doi.org/10.1002/ccr3.72376.